# iChip increases the success of cultivation of TBT-resistant and TBT-degrading bacteria from estuarine sediment

**DOI:** 10.1007/s11274-022-03297-2

**Published:** 2022-08-11

**Authors:** A. Polrot, J. R. Kirby, F. J. Olorunniji, J. W. Birkett, G. P. Sharples

**Affiliations:** Liverpool John Moores University, Byrom Street, Liverpool, L3 3AF UK

**Keywords:** Bacterial isolation, Bioremediation, High throughput isolation, Isolation chip, Tributyltin

## Abstract

Standard methods of microbial cultivation only enable the isolation of a fraction of the total environmental bacteria. Numerous techniques have been developed to increase the success of isolation and cultivation in the laboratory, some of which derive from diffusion chambers. In a diffusion chamber, environmental bacteria in agar medium are put back in the environment to grow as close to their natural conditions as possible, only separated from the environment by semi-permeable membranes. In this study, the iChip, a device that possesses hundreds of mini diffusion chambers, was used to isolate tributyltin (TBT) resistant and degrading bacteria. IChip was shown to be efficient at increasing the number of cultivable bacteria compared to standard methods. TBT-resistant strains belonging to *Oceanisphaera* sp., *Pseudomonas* sp., *Bacillus* sp. and *Shewanella* sp. were identified from Liverpool Dock sediment. Among the isolates in the present study, only members of *Pseudomonas* sp. were able to use TBT as a sole carbon source. It is the first time that members of the genus *Oceanisphaera* have been shown to be TBT-resistant. Although iChip has been used in the search for molecules of biomedical interest here we demonstrate its promising application in bioremediation.

## Introduction

Tributyltin is an organotin compound that has been used widely as a biocide in antifouling paints. It is therefore highly toxic and has been shown to be a major threat to aquatic ecosystems. Due to its toxicity, it was subjected to a global ban in 2008 (Sonak et al. 2009). However, TBT is still a major concern in many locations around the world (Filipkowska and Kowalewska 2019). In fact, it is still authorised in a small number of countries (Turner and Glegg 2014), and it is suspected to be used illegally in many others because of its high efficiency (Egardt et al. 2017). The main concern is its high persistence in anoxic sediments and as such, is a pernicious legacy contaminant. Indeed, TBT is hydrophobic and strongly binds to organic matter and sediment where it can remain for decades (Langston et al. 2015). Sediment therefore acts as a secondary source of contamination during resuspension events, causing more disturbance to aquatic ecosystems. There is therefore a need to remediate sediment contaminated with TBT.

Traditional remediation techniques such as incineration (Song et al. 2005) or electrochemical oxidation (Beuselinck and Valle 2008) are usually regarded as efficient but costly. In addition, they can cause environmental issues as they involve the excavation of sediment, which causes problems of contaminant spreading and further pollution due to carbon emissions during transportation (Manap and Voulvoulis 2015). The more environmentally sustainable approach is bioremediation, where contaminants are broken down by the activity of biological organisms. In particular, in situ bioremediation removes the need for excavation plus the associated cost and environmental issues linked to it (Polrot et al. 2021). Bioremediation can be further subdivided into phytoremediation, when using plants (Pilon-Smits 2005), or biodegradation, when using microorganisms (Adams et al. 2015). The latter is especially pertinent for in situ bioremediation of port sediment. Biodegradation includes natural attenuation, biostimulation and bioaugmentation (Tyagi et al. 2011; Adams et al. 2015). Natural attenuation consists of using the native microbial community to naturally degrade harmful contaminants (Lofrano et al. 2017). Biostimulation aims at boosting the degrading activity of the microbial community by providing more favourable conditions, for example by the addition of nutrients (Adams et al. 2015), or through oxygenation (Scow and Hicks 2005). Finally, bioaugmentation consists of adding specific microorganisms to decontaminate the material (Tyagi et al. 2011; Adams et al. 2015). The added microorganisms are selected for their exceptional abilities to efficiently degrade the contaminants of interest.

The use of bioremediation requires a comprehensive understanding of the degradation pathways and kinetics, the microbial communities involved in the degradation as well as the most favourable conditions for the growth and degrading activity of the microorganisms involved. A first step towards this objective is to proceed with the isolation and cultivation of the microbial degraders. Thus, research has been carried out to isolate and characterise TBT-resistant and degrading microorganisms (Cruz et al. 2015). Among the identified microbes include *Chlorella* species (Tsang et al. 1999; Jin et al. 2011) and fungi such as *Cunninghamella elegans* or *Cochliobolus lunatus* (Bernat and Długoński 2002; Bernat et al. 2013). In addition, many bacteria have been studied for their TBT degradation ability, such as *Aeromonas molluscorum*, *Enterobacter cloacae* and numerous species of *Pseudomonas* (Finnegan et al. 2018).

Despite this, it is well-known that only a small proportion of microbes have been discovered so far. Indeed, it is estimated that more than 99% of bacteria remain unknown (Locey and Lennon 2016). The main reason for this is our inability to cultivate them in the laboratory. Classic methods of isolation and cultivation, that were used for the isolation of TBT-degrading bacteria so far, failed to provide the appropriate conditions for the growth of the majority of the environmental bacteria and are biased towards the same species. Nevertheless, some techniques have been developed to improve the success of cultivation of novel species, usually by mimicking as accurately as possible the natural environment (Hahn et al. 2019; Bodor et al. 2020). Among these, the diffusion chamber concept was of special interest. In diffusion chambers, microorganisms are trapped in agar while in contact with their natural environment with semipermeable membranes. The membranes ensure that cells cannot move in or out of the diffusion chamber but small molecules that may be necessary for microbial growth can enter the chamber (Kaeberlein et al. 2002). On the basis of this concept, iChip was created, acting like hundreds of mini diffusion chambers and therefore allowing the high-throughput isolation of bacteria (Nichols et al. 2010). IChip allowed the cultivation of different species of bacteria than standard plating methods (Nichols et al. 2010).

The first aim of this study was to evaluate the beneficial potential of using iChip for the isolation of bacteria of interest in the field of bioremediation and more specifically for TBT biodegradation. A second aim was to advance the knowledge on TBT biodegradation in estuarine sediment with the isolation of TBT-resistant and TBT-degrading bacteria. To fulfil these objectives, a comparison of the standard plating and iChip techniques was performed by measuring the difference in culturability of sediment bacteria using the two techniques. TBT-resistant/degrading bacteria were then screened among the obtained isolates.

## Material and methods

### Sediment sampling and preparation

Sediment samples (textural class ‘slightly sandy mud’ (Flemming 2000), comprising 14.3% clay, 79.5% silt, 6.2% sand) were taken from Liverpool Brocklebank Dock. The samples had a pH of 7.8, salinity of 27 psu, total nitrogen content of 0.26%, total carbon content of 3.92% and total organic carbon (TOC) content of 3.12%. Sediment from Liverpool port was chosen for this study because TBT hotspots are usually concentrated around ports and harbours (Filipkowska and Kowalewska 2019). Sampling locations in the docks were chosen according to TBT contamination data from 2010 (data provided by Peel Ports). Organotin measurement revealed that the contamination in these samples was below detection level at the time of the sampling. This supports the hypothesis that the local microbial community is capable of TBT biodegradation and those samples were therefore selected for the present study. One sample remained untouched in a cold room, stored in the dark at a temperature of 4 °C. For microcosm experiments measuring TBT biodegradation in different environmental scenarios (Polrot 2022), another sample was sieved at 2 mm and spiked with 10 µg TBTCl/g dw sediment (concentration corresponding to a heavy contamination scenario and constrained by the detection limit of the organotin measurement method used) and thoroughly mixed by hand before being put back in the cold store for 4 weeks as an equilibration step. After that equilibration step, the mud was incubated at 20 °C for 3 months. At the end of this incubation period, the sample was used for the present study and is referred as “prepared sediment” for the rest of this paper. When using sediment stored directly after sampling and not processed further, the term “untouched sediment” is used.

### Sediment dilution and standard plating

Serial dilutions of the two types of sediment were plated on Tryptic Soy Agar (TSA) and TSA + 1 mM TBT in order to calculate the abundance of bacteria capable of growth in standard laboratory conditions. After inoculation of different sediment dilutions in triplicates, the agar plates were incubated at room temperature for 3 to 5 days before the enumeration of colonies was performed.

The result of this enumeration was used to calculate the appropriate dilution for the inoculation of one “cultivable” bacterial cell in 10% of the iChip through-holes (10^2^ bacteria per mL).

### iChip assembly and incubation

**Fig. 1:**
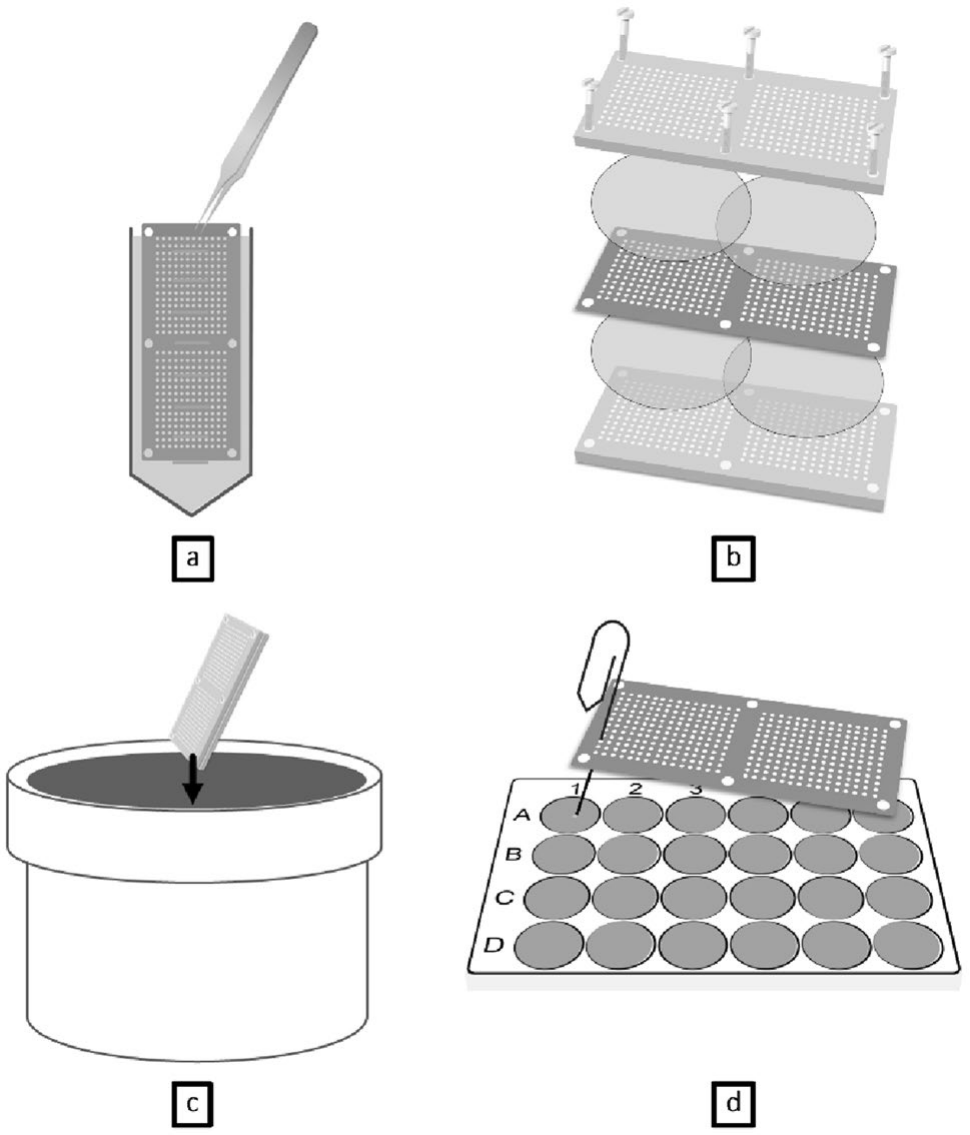
Steps to sediment bacteria isolation and cultivation using an iChip. The central plate is loaded with fusion agarose medium inoculated with sediment bacterial dilution (**a**). The iChip is then assembled with 0.03 µm polycarbonate membranes and the external plate, screwed together (**b**), and immerged in a bucket of muddy sediment for 2 weeks (**c**). After incubation, the iChip is thoroughly rinsed with sterile water, disassembled and sterile gauge clips are used to deposit each agar plug in a well of a 24-well plate filled up with TSA (**d**)

IChips were manufactured in the general engineering workshops of Liverpool John Moores University using the instructions provided by Nichols et al. (2010). Figure 1b indicates all of the components of an iChip, the central plate and the two external ones, which are pierced with a multitude of through-holes arranged in two arrays, in this case two arrays of 192 through-holes. Before assemblage, all the components were sterilized by immersion in 70% ethanol for 15 min. They were then allowed to dry under a sterile hood after which the central plate was immersed in molten agar (Fig. 1a) containing the appropriate sediment dilution as a means to load one cultivable bacterial cell in 10% of the through-holes (10^2^ cultivable cells/mL). Once the agar solidified on the central plate, the excess was removed using a sterile microscope slide and 8 sterile polycarbonate membranes disks of 27 cm diameter with 0.03 µm diameter pores were placed on each side. The external plates were finally mounted at the bottom and top of the central plates and the whole assemblage was screwed together (Fig. 1b). To avoid any leaking from the sides, petroleum jelly was applied to seal the edges of the iChip, which was then protected with a fine band of parafilm. After assemblage, the iChips were immersed in a bucket of sediment and stored at 20 °C for a week (Fig. 1c).

### Isolate recovery

After the incubation period, the iChips were thoroughly rinsed in sterile distilled water and disassembled. About one hundred random cores were retrieved from each iChip using a sterile and unbound gauge paper clip and gently crushed on the surface of TSA medium in 24-well plates (Fig. 1d). The 24-well plates were incubated for several weeks at room temperature in the dark. The percentage of positive wells at this step was used to calculate the difference in cultivability between iChip and standard plating.

### Screening for TBT resistance and use as sole carbon source

Each isolate that could be grown on the 24 well plates containing TSA from the iChip cores were subcultured on TSA + 1 mM TBT to screen for the resistance phenotype.

The isolates that could grow on TSA + 1 mM TBT were further subcultured on Minimal Salt Medium (MSM) containing 1 mM TBT as the sole carbon source. MSM was prepared with the following compounds per litre of distilled water: 0.06 g ferrous sulphate; 12.6 g dipotassium hydrogen orthophosphate; 3.64 g potassium dihydrogen orthophosphate; 2 g ammonium nitrate; 0.2 g magnesium sulphate; 0.0012 g sodium molybdate; 0.0012 g manganese sulphate; 0.15 g calcium chloride; 15 g agar. 1L of medium containing only agar and the phosphate buffer was autoclaved, all the other elements were prepared in solution separately, filter sterilized and added to the fusion medium after autoclaving and before pouring into petri dishes.

### Genus identification of the isolates

#### DNA extraction

24 colonies growing on TSA + 1 mM TBT were selected to be further identified by 16S rRNA gene sequencing. 20 isolates coming from the isolation through iChip, and 4 isolates obtained using the classic method of isolation. Freshly grown colonies were resuspended in 30 µL of sterile water and heated at 95 °C for 10 min to extract their DNA. The suspensions were then spun down for 2 min in a benchtop centrifuge at maximum speed and the supernatant was used as template DNA for the PCR steps.

#### DNA amplification

The amplification was performed using the following universal primers: 27F (AGAGTTTGATCATGGCTCA) and 1492R (TACGGTTACCTTGTTACGACTT). The reaction was prepared in a volume of 50 µL in total, with 25 µL of ReadyMix™ (Sigma), 1 µL of 10 pM of each primer and 2µL of DNA. Reactions were then performed in a thermocycler with the following program: 94 °C for 2 min of initial denaturation followed by 35 cycles at 94 °C for 1 min, 58 °C for 30 s and 72 °C for 1 min, finishing with a final extension at 72 °C for 10 min. The amplification of the samples was detected along with a DNA molecular weight standard (1 kb + , Invitrogen) by electrophoresis in a 2% agarose gel stained with SYBR Safe (Invitrogen) and visualized by transillumination by UV light.

The DNA concentration was then measured using a Nanodrop. As all the concentrations were too low, the samples were evaporated and resuspended in the appropriate volume to obtain 25 ng/µL. 5 µL of each sample was then added to 5 µL of primer at 5 pmol/µL. 24 tubes were prepared with the forward primer 27F and 24 others with the reverse primer 1492R. The 48 tubes were barcoded using the LightRun barcodes from Eurofins Genomics and sent to the company for Sanger sequencing.

#### Sequence analyses

The ab1 files received from Sanger sequencing were checked for quality and the sequences appropriately corrected. The forward and reverse sequences of the same isolates were aligned and reassembled using BioEdit and the resulting FASTA sequences were analysed by BLAST using the total database, excluding uncultured/environmental sample sequences.

#### Nucleotide sequence accession number

The sequences were deposited in GenBank and their accession numbers are detailed in Table 1.

**Table 1:** Details of the isolates identified by Sanger sequencing of the 16S rRNA genes

Accession number of 16S rRNA gene sequence	Isolate	Isolation technique used	Growth on the following medium after 4th subculturing			Identification
			TSA	TSA + 1 mM TBT	MSM + 1 mM TBT	
OM158192	β2A3	iChip—prepared sediment	+	+	+	*Pseudomonas* sp.
OM158193	β2B2	iChip—prepared sediment	+	+	+	*Pseudomonas* sp.
OM158197	β5A5	iChip—prepared sediment	+	+	+	*Pseudomonas* sp.
OM158198	β5A6	iChip—prepared sediment	+	+	+	*Pseudomonas* sp.
OM158201	β5C4	iChip—prepared sediment	+	+	+	*Pseudomonas* sp.
OM158202	β5C5	iChip—prepared sediment	+	+	+	*Pseudomonas* sp.
OM158200	β5C3	iChip—prepared sediment	+	+	+	*Pseudomonas* sp.
OM158183	3A1	standard plating	+	+	+	*Pseudomonas* sp.
OM158184	3A2	standard plating	+	+	+	*Pseudomonas* sp.
OM158203	I13b	standard plating	+	+	+	*Pseudomonas* sp.
OM158191	α4D6	iChip—prepared sediment	+	+	−	*Oceanisphaera* sp.
OM158190	α4A2	iChip—prepared sediment	+	+	−	*Oceanisphaera* sp.
OM158189	α3D4	iChip—prepared sediment	+	+	−	*Oceanisphaera* sp.
OM158187	α1C3	iChip—prepared sediment	+	+	−	*Oceanisphaera* sp.
OM158185	7A	standard plating	+	+	−	*Pseudomonas* sp.
OM158186	α1B6	iChip—prepared sediment	+	−	−	*Oceanisphaera* sp.
OM158188	α1D5	iChip—prepared sediment	+	−	−	*Oceanisphaera* sp.
OM158195	β2C5	iChip—prepared sediment	+	−	−	*Pseudomonas* sp.
OM158194	β2B6	iChip—prepared sediment	+	−	−	*Pseudomonas* sp.
OM158196	β2D5	iChip—prepared sediment	+	−	−	*Pseudomonas* sp.
OM158199	β5B5	iChip—prepared sediment	+	−	−	*Pseudomonas* sp.
OM158204	γ1D4	iChip—prepared sediment	+	−	−	*Bacillus* sp.
OM158206	Z3D5b	iChip—untouched sediment	+	−	−	*Shewanella* sp.
OM158205	Z3D5a	iChip—untouched sediment	+	−	−	*Shewanella* sp.

This table describes the different isolates and the techniques used for their obtention as well as their growth capacities when the identification was performed and the result of the identification. All of these isolates could grow on TSA + 1 mM at the 1st subculturing

### Statistical analyses

All statistical analyses were performed using R Studio. Significant differences in the cultivability of bacteria using the two methods were calculated with a Student’s t-test. Statistical significance was assumed when the p-value was below or equal to 0.05.

## Results

### Abundance of cultivable bacteria on TSA medium

**Fig. 2:**
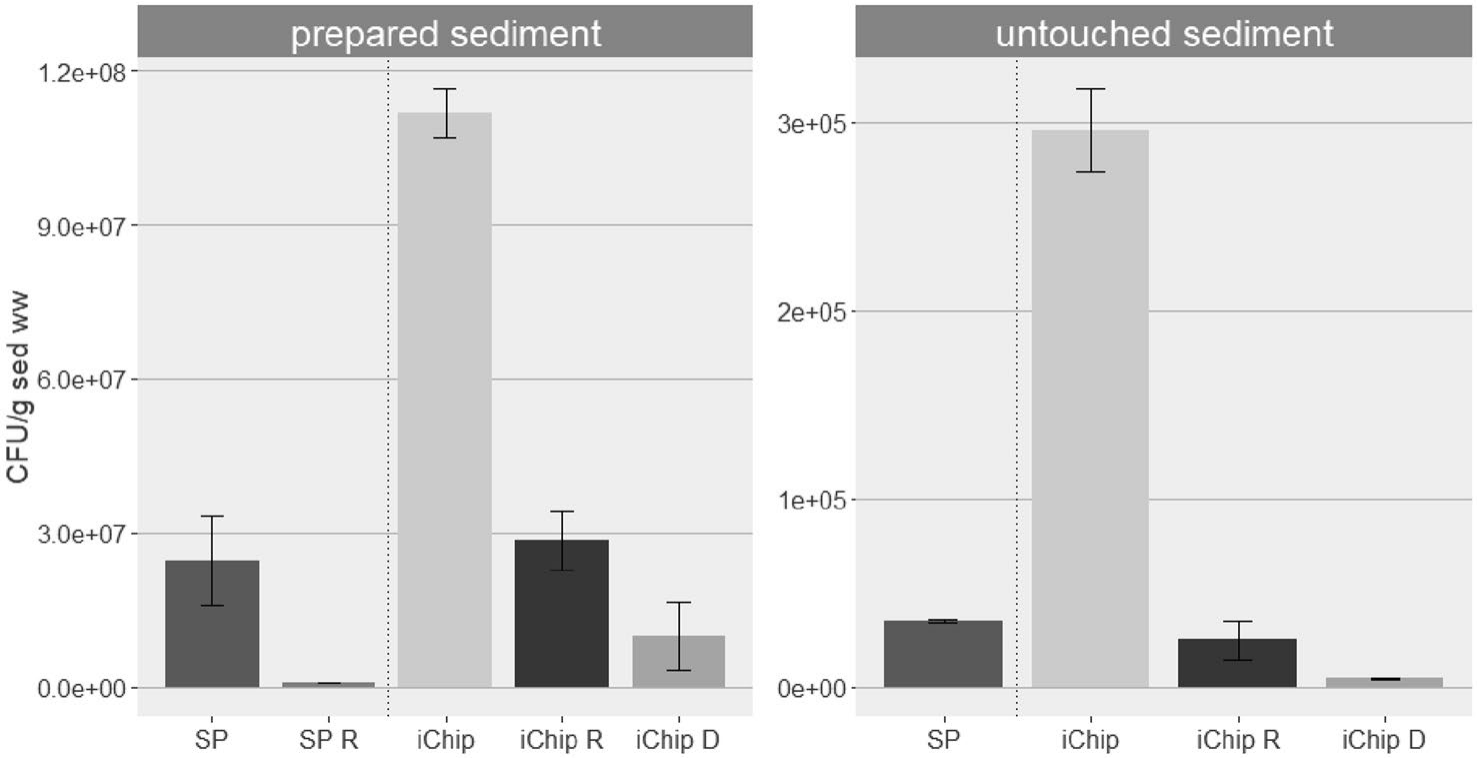
Difference in cultivability between standard plating and iChip method using prepare or untouched sediment. SP: CFU numbers obtained by Standard Plating; SP R: TBT-resistant CFU numbers obtained by Standard Plating on TSA + 1 mM TBT; iChip: CFU obtained after one round of iChip and subculturing on TSA; iChip R: TBT-resistant CFU numbers from the subculturing of isolates coming from iChip; iChip D: CFU numbers for cells able to use TBT as sole carbon source from the subculturing of isolates coming from iChip. Results shown represent the mean of triplicates and the error bars are the standard deviations

The abundance of cultivable bacteria increased significantly (prepared sediment: p value = 0.003, native sediment: p value = 0.007) when using one round of culturing in iChip compared to standard plating on TSA plates (Fig. 1). The number of CFU increased by a factor of 5.5 and 9.5 for the experiment involving untouched sediment and prepared sediment respectively (Fig. 2). A higher abundance of cultivable bacteria was also observed for the method using prepared sediment compared to untouched sediment.

### Proportion of TBT-resistant bacteria cultivated using iChip compared to standard plating

From untouched sediment, no TBT-resistant bacteria could be grown using the standard method of plating sediment dilutions on TSA in petri dishes. However, the proportion of TBT-resistant bacteria among the isolates firstly grown on TSA without TBT was not checked. Note as well that no isolates could be obtained when preparing an iChip using TSA containing 1 mM TBT.

Nevertheless, TBT-resistant proportions among iChip isolates on TSA using the two different types of sediment can be compared. A higher proportion of TBT-resistant bacteria was found for prepared sediment (p value = 0.038), with 38.2% of TBT-resistant isolates obtained from prepared sediment compared to 16.3% for untouched sediment (Fig. 2).

### Proportion of bacteria using TBT as sole carbon source

Although the mean number of bacteria capable of using TBT as the sole carbon source appeared higher for prepared sediment compared with untouched sediment (9.3% and 2.0% of the isolates respectively), no statistical difference could be detected due to the high variability within the triplicates (Fig. 2, p value = 0.36).

### Identification of the isolates through 16S rRNA genes Sanger sequencing

After several rounds of cultivation, some isolates could not be recovered. The remaining isolates growing on TSA after four rounds of cultivation were therefore further identified. 18 of them came from iChip experiment using prepared sediment, two came from iChip experiment using untouched sediment and four came from standard plating isolation (Table 1).

As shown in Table 1, after the Sanger sequencing of 16S rRNA genes, four distinctive genera were identified by BLAST analyses: *Pseudomonas *sp.; *Shewanella* sp.; *Bacillus* sp. and *Oceanisphaera* sp. All of them were able to grow on TSA + 1 mM TBT for at least four subculturing attempts. However, some of them stopped growing after this 4^th^ step, but they could still grow on TSA without TBT.

The four isolates coming from standard plating were identified as *Pseudomonas* sp. In the names of the isolates, the first letter represents the label of an iChip (α, β, γ, Y). A correlation seems to be observed between the isolate’s genera and the iChip experiment.

## Discussion

### IChip increases the abundance of culturable bacteria

The period of culturing in iChip constitutes a good adaptation step prior to growth of bacteria on synthetic media. While a bacterium is trapped in TSA in an iChip buried in sediment, molecules that may be necessary for their growth can diffuse across the polycarbonate membranes and into the medium. As the growing conditions are closer to those of the natural environment, it is not surprising greater cultivation success is achieved. The real benefit of using the technique is the fact that after sub-culturing iChip agar plugs on TSA in full laboratory conditions, a greater variety of bacteria are able to grow compared to the attempts at isolation without using the intermediate step in iChip.

The mechanisms behind this adaptation are unclear. It is also important to note that among the initial isolates which could grow after the direct subculturing from iChip, a number of others failed to grow after a couple of subculturing attempts. As our interest was focused on TBT resistant bacteria, only these were subcultured. Failure to maintain bacterial isolates after subculturing is often described but there are a lack of explanations for this issue (Overmann et al. 2017; Hahn et al. 2019). As the subculturing was performed on TSA + 1 mM TBTCl, some hypotheses can be proposed to explain this lack of growth, in addition to an unknown cause. First, the subculturing may have been delayed, and the bacteria could not be recovered after being kept in the fridge for a few weeks. Second, during the subculturing, a very small quantity of key molecules necessary for the growth of some isolates may have been utilised during the initial subculturing stages but eventually became depleted. Finally, given the selectivity of the medium used, the bacteria could simply have lost their ability to grow in the presence of TBT. This explanation was confirmed for some of the isolates, which after the fourth subculturing stage could be grown on TSA but not on TSA + 1 mM TBTCl. This loss of resistance is most likely to occur through the loss of a plasmid, therefore suggesting that the resistance genes are located on a plasmid for at least some of these strains. Plasmid loss is a well-studied phenomenon due to the wide use of plasmids in research but our understanding remains incomplete (Carroll and Wong 2018). Plasmids are usually well maintained in the presence of a selective pressure, here TBT, but if the isolation plates are kept long enough for TBT degradation to occur, the selective pressure could be reduced around the isolates, which would increase the chance of plasmid loss (Hanak and Cranenburgh 2001).

### A higher proportion of TBT-resistant bacteria are found among isolates obtained from prepared sediment

In the literature, bacteria are usually called resistant when growing on a medium containing a biocide concentration that kills 90% of the population (Cruz et al. 2015). For the purpose of this study, however, TBT-resistant bacteria are those bacteria that grow on a medium containing 1 mM TBTCl. Observing a higher proportion of TBT-resistant bacteria among the isolates obtained from prepared sediment compared to the ones obtained from untouched sediment is to be expected.

Different mechanisms can lead to bacterial resistance to TBT. There are at least four theoretical categories of resistance mechanism: (1) TBT exclusion/efflux from the cell; (2) TBT degradation into DBT, MBT and inorganic tin; (3) TBT metabolization and use as a carbon source and (4) bioaccumulation using metallothionein-like proteins (Cruz et al. 2015). Determining the resistance mechanism used by the bacteria isolated in this study would require further testing. Previous studies of TBT-resistant bacteria have been able to identify some genes and molecules involved in the resistance mechanisms. Transcriptomic studies have looked at the difference in gene expression in the presence of TBTCl. Bernat et al. (2014) reported a clear change in membrane phospholipid composition as well as production of peroxidase. The peroxidase could have a protective role against the generation of reactive oxygen species that have been reported to play a critical role in TBTCl toxicity. Efflux pumps have been identified as a basis of the resistance in two bacterial species, coded by the operon *tbtABM* in some *Pseudomonas stuzeri* strains (Jude et al. 2004) and coded by the gene *SugE* in *Aeromonas molluscorum* (Cruz et al. 2013).

For a bioremediation purpose, the mechanism of most interest is the degradation of the compound. A quick way of checking for degradation ability is to provide TBT as sole carbon source in the growth medium. Therefore, further tests were carried out to identify this type of TBT-degrader among the isolated strains.

### Some of the isolates are able to use TBT as the sole carbon source

As a straightforward way of screening TBT-degrading bacteria, the TBT-resistant isolates were subcultured on a medium containing TBT as the sole carbon source. Growth on this medium demonstrates the ability of the bacteria to use TBT as a sole carbon source.

The high variability of the results prevented the detection of a statistical difference between the proportion of isolates able to use TBT as sole carbon source in prepared sediment and untouched sediment. A higher number of bacteria using TBT as the sole carbon source in the prepared sediment would be an expected result as the presence of TBT will have favoured a population of bacteria that was adapted to the presence of such a biocide. TBT degradation and its use as a carbon source is thought to happen through sequential debutylation but the enzymes responsible for this degradation have never been clearly identified (Cruz et al. 2015). In Hassan (2017), the author suggests a role of the protein sugE in TBT degradation as its overexpression enhanced TBT degradation, but the addition of the gene *sugE* alone could not provide the degradation phenotype in *E. coli*. In parallel, siderophores produced by *Pseudomonas chlororaphis* have been shown to be responsible for Tin-C cleavage using triphenyltin (TPT), diphenyltin (DPT) and dibutyltin (DBT) as the substrates and may have the same effect on TBT (Inoue et al. 2003). For siderophores, as well as enzymatic degradation, however, TBT may not be the intended target and its degradation could result from co-metabolism. It is important to emphasise that bacteria, which are not able to use TBT as the sole carbon source could still have the ability to degrade it. Further tests would be necessary to resolve this.

### iChip reveals members of *Oceanisphaera*, *Bacillus*, *Shewanella* and *Pseudomonas* as TBT-resistant bacteria, and members of *Pseudomonas* as TBT-degrading bacteria

The loss of the resistance ability for some of the isolates after a couple rounds of subculturing on TSA + 1 mM TBT would suggest a plasmidic location of the resistance genes. These include the only *Bacillus* sp. isolate, the two *Shewanella* sp. isolates, some of the *Pseudomonas* sp. and *Oceanisphaera* sp. isolates.

The remaining *Oceanisphaera* sp. isolates were still maintained on TSA + 1 mM TBT but could not grow on MSM + 1 mM TBT, which means that they were not capable of using TBT as the sole carbon source. At this stage it cannot be determined if they are still capable of TBT degradation by another mechanism. TBT could be degraded by an adverse reaction of enzymes secreted by the bacteria without utilisation of the degradation product. Nevertheless, this is the first time that members of the genus *Oceanisphaera* have been shown to be capable of TBT resistance. *Oceanisphaera* members have been repeatedly isolated from coastal and marine sediment (Romanenko et al. 2003; Shin et al. 2012; Zhou et al. 2015; Cho and Lee 2016), the present study therefore shows their presence in estuarine sediment too.

Finally, many of the isolates belonging to *Pseudomonas* sp. were able to use TBT as the sole carbon source. This result is not surprising as *Pseudomonas* members have often been reported as TBT-resistant and as TBT-degraders (Roy et al. 2004; Khanolkar et al. 2015; Yáñez et al. 2015; Ebah et al. 2016). In addition they are also known to degrade a wide range of other sediment contaminants (Wasi et al. 2013).

It is interesting to note that all of the isolates coming from the same iChip experiments belong to the same genera, although the small numbers of representatives for some iChips prevent statistically significant conclusions to be made.

### Discussion on the use of iChip for the isolation of uncultured bacteria

Owing to its design, iChips are useful tools for the high throughput isolation of bacteria from a wide range of environments. In iChip, bacterial cells can easily be isolated from one another, and their growth is facilitated by the close proximity to the environment. One of the issues stated for the cultivation of unknown bacteria is that the fast-growing species outcompete the slow growing or rare species on the culture plates but in iChips, each bacterial cell occupies one of the many through holes, giving more chance for these species to successfully develop. IChip, however, will not solve every issue. For example, the subculturing is later done in full laboratory conditions, and as this paper shows, not all the bacteria that have been able to grow in iChip are adapted for further growth on synthetic medium. Ideally, a coupling of iChip and the use of alternative media and growth conditions could lead to the best results. The need for key growth factors that are normally not present in the classic incubation media may persist after subculturing out of the iChip, and media supplemented with different types of molecules would still be useful. On the contrary, the nutrient-rich media classically used have sometimes been pointed out as inhibitory to some types of bacteria referred to as ‘oligophilic ‘ which would only develop on nutrient-poor media (Watve et al. 2000). Lowering the temperature of incubation is also usually suggested and this was done in the present study where all the incubation steps were performed at 20 °C.

## Conclusion

iChip was previously shown to successfully increase the success of cultivation of bacteria producing metabolites of medical interest (Piddock 2015) and here we demonstrated its efficiency in increasing the abundance of culturable bacteria of interest in the field of bioremediation and more specifically TBT biodegradation. Further effort is however required in order to maintain most of these isolates in full laboratory conditions after the steps of growth in iChips. After identification of the isolates obtained by iChip, members of the genus *Oceanisphaera* were found associated with TBT resistance for the first time.

## Data Availability

The datasets generated during and/or analysed during the current study are available from the corresponding author on reasonable request.
